# Protective effects of *Descurainia sophia* seeds extract and its fractions on pulmonary edema by untargeted urine and serum metabolomics strategy

**DOI:** 10.3389/fphar.2023.1080962

**Published:** 2023-02-14

**Authors:** Jinying Zhang, Ning Zhou, Yongxiang Wang, Tong Liu, Yumin Cao, Weisheng Feng, Xiaoke Zheng

**Affiliations:** ^1^ College of Pharmacy, Henan University of Chinese Medicine, Zhengzhou, China; ^2^ The Engineering and Technology Center for Chinese Medicine Development of Henan Province, Zhengzhou, China; ^3^ Co-construction Collaborative Innovation Center for Chinese Medicine and Respiratory Diseases by Henan, Education Ministry of P.R, Zhengzhou, China

**Keywords:** *Descurainia sophia* seeds, split fractions, pulmonary edema, metabolomics, UHPLC-Q/TOF-MS, biomarkers

## Abstract

**Background:** *Descurainia sophia* seeds (DS) is a herbal medicine in traditional Chinese medicine (TCM) for treating lung diseases. We aimed to evaluate the therapeutic effect of DS and five of its fractions upon pulmonary edema (PE) through metabolomics analysis (MA) of urine and serum samples of rats.

**Methods:** A PE model was established by intrathoracic injection of carrageenan. Rats were pretreated with DS extract or its five fractions (polysaccharides (DS-Pol); oligosaccharides (DS-Oli); flavonoid glycosides (DS-FG); flavonoid aglycone (DS-FA); fat oil fraction (DS-FO)) for seven consecutive days. Forty-eight hours after carrageenan injection, lung tissues were subjected to histopathology. MA of urine and serum was done by ultra-high performance liquid chromatography-quadrupole time-of-flight mass spectrometry, respectively. Principal component analysis and orthogonal partial least squares-discriminant analysis were operated for the MA of rats and potential biomarkers related to treatment. Heatmaps and metabolic networks were constructed to explore how DS and its five fractions act against PE.

**Results:** DS and its five fractions could all attenuate pathologic lung injury to different degrees, and DS-Oli, DS-FG, and DS-FO had a more potent effect compared with DS-Pol and DS-FA. DS-Oli, DS-FG, DS-FA, and DS-FO could regulate the metabolic profiles of PE rats, but DS-Pol was less potent. According to MA, the five fractions could improve PE to some degree due to their anti-inflammatory, immunoregulatory, and renoprotective activities by mediating the metabolism of taurine, tryptophan, and arachidonic acid. However, DS-Oli, DS-FG, and DS-FO had more important roles in edema-fluid reabsorption, and reduction of vascular leakage through regulating the metabolism of phenylalanine, sphingolipid and bile acid. Finally, heatmaps and hierarchical clustering analysis indicated DS-Oli, DS-FG, and DS-FO to be more efficacious than DS-Pol or DS-FA against PE. The five fractions of DS had a synergistic effect on PE from different aspects, thereby constituting the entire efficacy of DS. DS-Oli, DS-FG, or DS-FO could be used as an alternative to DS.

**Conclusion:** MA combined with use of DS and its fractions provided novel insights into the mechanism of action of TCM.

## 1 Introduction

Pulmonary edema (PE) is a main pathologic characteristic in the progression of acute lung injury, acute respiratory distress syndrome, and severe pneumonia. (Birukov et al., 2013). The pathogenic mechanism of PE is complicated, mainly involving increased endothelial permeability, hampered clearance of alveolar liquid, as well as accumulation of extravascular fluid in the lung interstitium and alveoli (Ingbar, 2019). Recently, it was reported that acute lung edema is a common manifestation of heart failure (Clark and Cleland, 2013). Therefore, characterized by the high prevalence of morbidity and mortality, PE seriously threatens human health. Due to its complex etiology, few efficacious therapies are available. (Ingbar, 2019; Cui et al., 2021).

In recent years, traditional Chinese medicine (TCM) have received increasing attention worldwide due to their excellent efficacy and low toxicity (Hsieh et al., 2020). *Descurainia sophia* (L.) Webb.ex Prantl, also known as “flixweed”, is a member of the Brassicaceae family (Zhou et al., 2017). The dry ripe seed of *D. sophia* (DS; known as “Ting Li Zi” in Chinese) is a common herbal medicine used in northeast Asia (Kim, S. B. et al., 2019) to cure pulmonary disorders caused by phlegm-fluid retention (e.g., asthma, cough, edema) (Baek et al., 2018; Luo et al., 2018; Ting et al., 2019). However, studies on its therapeutic effect on pulmonary diseases are scarce.

In view of the complexity of its chemical composition, an aqueous extract of DS can be split into five fractions according to polarity and chemical structure: polysaccharides (DS-Pol), oligosaccharides (DS-Oli), flavonoid glycosides (DS-FG), flavonoid aglycones (DS-FA), and fat oil (DS-FO) (Zhou, et al., 2017). Our previous pharmacodynamic studies revealed that DS and its five fractions could (to some degree) mitigate PE. We found that DS-Oli, DS-FG, and DS-FO fractions had a more robust therapeutic effect than DS-Pol and DS-FA fractions. However, detection of some biochemical indices and signaling pathways cannot reveal the overall effects of DS.

Metabolomics analysis (MA) can provide a powerful approach for exploring the complex mechanisms of multi-constituent TCM through comprehensive analysis of associated metabolites in biological samples (urine, serum, tissue, feces) (Zhou, N. et al., 2021). Recently, MA has been employed increasingly to investigate the relationship between the components of TCM and diseases from a holistic perspective (Li et al., 2022; Wang, D. et al., 2019; Zhou et al., 2019).

Here, untargeted MA using urine and serum was conducted to investigate the therapeutic effect and possible metabolic regulatory mechanism of DS and its five fractions in PE treatment. First, a carrageenan-induced PE model was established to simulate the pathologic state of PE, and histopathology was undertaken to evaluate the model and treatment effects of DS and its fractions. Then, untargeted MA using urine and serum based on ultra-high performance liquid chromatography-quadrupole time-of-flight mass spectrometry (UHPLC-QTOF-MS) was undertaken to assess metabolic-profile changes and identify differential metabolites through multivariate analysis. Finally, a metabolic network was constructed to further uncover the overall mechanism of action of DS, DS-Pol, DS-Oli, DS-FG, DS-FA, and DS-FO on PE and evaluate the effects of these five fractions.

## 2 Materials and methods

### 2.1 Chemicals and reagents

DS was purchased from Beijing Tongrentang Pharmacy (Beijing, China) and authenticated by Professor Sui-Qing Chen (Henan University of Chinese Medicine, Zhengzhou, China). The voucher specimen was deposited with our research team in Henan University of Chinese Medicine. Carrageenan (batch number: C1013-25G) was purchased from MilliporeSigma (Burlington, MA, United States). Dexamethasone (DEX: batch number: 180102) was obtained from Xianjv Pharmaceuticals (Zhejiang, China). LC-MS-grade acetonitrile and formic acid (FA) of purity 99% were obtained from Thermo Fisher Scientific (Waltham, MA, United States).

### 2.2 Preparation of DS extract and its five fractions

The dried DS were decocted twice with eightfold and tenfold distilled water at 100°C, respectively (1 h each time). Supernatants were combined, concentrated, and dried under a vacuum to obtain an aqueous extract of DS (extract ratio: 7.26%), which was extracted with petroleum ether to obtain DS-FO. Then, the residue was concentrated and eluted with water, 20% ethanol (*v/v*), and 80% ethanol (*v/v*) on a column (Diaion HP-20; MilliporeSigma) to obtain the water-elution fraction, 20% ethanol fraction (corresponding to DS-FG), 80% ethanol fraction (corresponding to DS-FA), respectively. Subsequently, the water-elution fraction was precipitated with 95% ethanol (*v/v*) to obtain the precipitate (DS-Pol) and supernatant (DS-Oli). All fractions were stored at 4°C. The extract ratio of the five fractions (DS-Pol/DS-Oli/DS-FG/DS-FA/DS-FO) were: 1.231%, 1.306%, 0.289%, 0.517%, 28.8%. DS and its fractions were isolated systematically, purified and identified accurately in the previous phytochemical studies described by Feng and his colleagues (Gong et al., 2014; Feng et al., 2015; Feng et al., 2016). The chemical profiles of DS and its fractions were characterized by our team through HPLC combined with three detectors (PDA/ELSD/UV) and LC-MS analysis to guarantee stability and reproducibility of the fraction-splitting method and non-overlapping property of different fractions (Zhang, 2015; Zhou et al., 2017).

### 2.3 Establishment of the PE model and administrating

Animal experiments were conducted according to the Guidelines for Care and Use of Laboratory Animals of Henan University of Chinese Medicine. The study protocol was approved (DWLL2018080003) by the Animal Ethics Committee of Henan University of Chinese Medicine.

Male Sprague–Dawley rats (6–7 weeks; 180–220 g) were purchased from Beijing Charles River Laboratories Animal Technology (animal license number = SCXK (Jing) 2019-0011; Beijing, China). Rats were maintained under standard laboratory conditions at 20°C ± 2°C and humidity of 60% ± 10%. They were exposed to a 12-h light–dark circle, and had free access to water and food.

Administrated dosages and establishment of the PE model were in accordance with our previous pharmacological experiment (Wang et al., 2022). Briefly, we previously explored gavage dose for rats based on 7 times (low dose, 1.167 g/kg/d), 14 times (middle dose, 2.334 g/kg/d) and 28 times (high dose, 4.668 g/kg/d) of traditional dose of DS (10 g/60 kg/d, crude herb) and determined middle dose (2.334 g/kg/d)as a suitable dose (i.e., 170 mg/kg/d, DS extract). Doses of all fractions were calculated according to their extract ratio from DS. After being allowed to acclimatize to their surroundings for 7 days, 72 rats were divided randomly into nine groups: normal control group (NC); PE model group (PE); DEX group (DEX, 0.075 mg/kg) and six DS-treatment groups, i.e., DS extract (170 mg/kg), DS-Pol (28.74 mg/kg), DS-Oli (30.48 mg/kg), DS-FG (6.75 mg/kg), DS-FA (12.07 mg/kg), and DS-FO (672.19 mg/kg).

Rats in the DEX group and six DS-treatment groups were pre-treated by gavage at 10 mL/kg per day for seven consecutive days. Rats in the NC group and model group received an equal volume of physiologic saline. Carrageenan was dissolved in 0.9% saline and intrathoracic injection was performed in all treatment-group rats at 20 mg/kg on day-7 to construct a carrageenan-induced PE model, whereas rats in the NC group were given an identical volume of 0.9% saline. Forty-8 hours after carrageenan intrathoracic injection, all rats were staying in metabolic cage for 12 h and obtained urine samples. Then, all rats were euthanized by injection of over-dose pentobarbital sodium and collected the whole blood from the abdominal aorta and lung tissue.

### 2.4 Histopathology analysis

Left lung lobes (three per group) were chosen randomly and fixed with paraformaldehyde for 24 h to prepare tissue slices and then stained with hematoxylin and eosin (H&E) for observation under an optical microscope (Eclipse TS100; Nikon, Tokyo, Japan). And lung histopathological manifestation was evaluated by the semi-quantitative scoring system (Zhu et al., 2020). Every variable (neutrophil infiltration, hemorrhage in alveoli and interstitial space, edema, atelectasis and necrosis) was scored from 0 to four scores based on the severity of lung injury. No injury scored 0, injury in 25% scored 1, injury in 50% scored 2, injury in 75% scored 3 and injury throughout the field score 4.

### 2.5 Preparation of urine and serum samples

Urine samples were stored at −80°C after collection. Before analyses, urine samples were thawed to room temperature and centrifuged (12,000 rpm, 10 min, 4°C). Then, urine (300 μL) was mixed thoroughly with acetonitrile (900 μL) on ice. The mixture was vortex-mixed for 1.5 min and centrifuged (12,000 rpm, 10 min, 4°C) to obtain the supernatant for UHPLC-MS injection.

Blood samples were allowed to stand for 30 min and centrifuged (3,000 rpm, 10 min, 4°C). The supernatant (serum) obtained was stored at −80°C. Before analyses, serum samples were thawed and each aliquot (100 μL) was diluted with acetonitrile (300 μL) on ice, vortex-mixed for 1.5 min, and centrifuged (12,000 rpm, 10 min, 4°C) to remove protein precipitates. The supernatant obtained was transferred to 2 mL vials for UHPLC-MS.

An aliquot (20 μL) from each urine sample and serum sample was mixed to provide quality control (QC) samples. Before the sample sequence, six continuous injections of QC were run. And QC samples were injected after eight samples had passed through the LC-MS system to monitor the stability and repeatability of the analytical system (Li et al., 2022).

### 2.6 UHPLC-Q/TOF-MS analysis of samples

Chromatography was carried out on an UHPLC system (Dionex Ultimate 3,000; Thermo Scientific, Waltham, MA, United States) equipped with an Acclaim™ RSLC 120 C18 chromatographic column (2.1 mm × 100 mm, 2.2 μm) and the column temperature was maintained at 40°C. The mobile phase comprised 0.1% formic acid–water (*v/v*, phase A) and acetonitrile (phase B) and the flow rate was 0.3 mL/min. Injection of each sample (2 μL) was analyzed after equilibration. The gradient elution program for urine samples was: 0–3 min, 5%–17% B; 3–17 min, 17%–18% B; 17–20 min, 18%–95% B; 20–22 min, 95%–5%; 22–26 min, 5%. The gradient elution program for serum samples was: 0–4 min, 10%–75% B; 4–12 min, 75%–80% B; 12–14 min, 80%–95% B; 14–16 min, 95%–10%; 16–19 min, 10%.

MS with an eletrospray ionization source (ESI) was carried out on a Q/TOF mass spectrometer (maXis HD; Bruker, Billerica, United States) in positive and negative ion modes to analyze urine and serum samples, respectively. The main MS parameters were: spectra rate = 1 Hz; scanning range of *m/z* = 50–1,500; capillary voltage = 3,500/3200 V (positive/negative ion modes); pressure of nebulizer gas = 2.0 bar; dry-gas temperature = 230°C; flow rate = 8 L/min.

### 2.7 Data processing and multivariate statistical analysis

Original data collected by the UPLC-QTOF-MS system were processed by Profile Analysis 2.1 (Bruker) for the recognition, calibration, alignment, and normalization of peaks, and then converted into TXT format. To acquire more complete metabolic profiles, the obtained data in the positive and negative modes were merged and imported into SIMCA-P 14.0 (Umetrics, Stockholm, Sweden) for multivariate statistical analysis.

Principal component analysis (PCA) is an unsupervised method of pattern recognition. PCA can reveal visually the natural grouping of samples (Xu et al., 2017; Hu et al., 2020). Each point represents a sample in a PCA score plot, which is convenient for the removal of outliers (Zhou et al., 2017). Whereas, orthogonal projections to latent structures discriminant analysis (OPLS-DA) is carried out for supervised regression modeling and identifies potential differential biomarkers between groups (Wang, T. et al., 2019). The values of R^2^Y and Q^2^ are key indices to evaluate the fitting quality and predictability of OPLS-DA models. The values of Q^2^ were larger than 0.5 and the difference between R^2^Y and Q^2^ was less than 0.3, suggesting superior quality of our models. (Wang, X. et al., 2020).

Variable importance in projection (VIP) is implemented to measure the contribution to sample classification (Yuan, Z. et al., 2020). Coupled with VIP values (VIP >3) and the Student’s t-test (*p* < 0.05), the *m/z* of significantly altered metabolites was filtered preliminarily. According to the retention time, an accurate molecular weight was determined through DataAnalysis 4.4 software (Bruker). And next, biomarker (error <5 ppm) were identified through the Human Metabolome Database (www.hmdb.ca) and Metaboanalyst (www.metaboanalyst.ca/) based on their retention time, accurate molecular weights and MS/MS fragments of ions (Zhou et al., 2019; Li et al., 2022). Hierarchical clustering analysis (HCA) of biomarkers was carried out and corresponding heatmaps were acquired using Mev 4.8.0 (MeV, United States). Finally, a network diagram of perturbed metabolic pathways in the PE model involving intervention by DS, DS-Pol, DS-Oli, DS-FG, DS-FA, or DS-FO was created using the Kyoto Encyclopedia of Genes and Genomes (KEGG; www.kegg.jp/), MetaboAnalyst, and MBRole (http://csbg.cnb.csic.es/mbrole2/) databases.

## 3 Result

### 3.1 Histopathological observation

**FIGURE 1:**
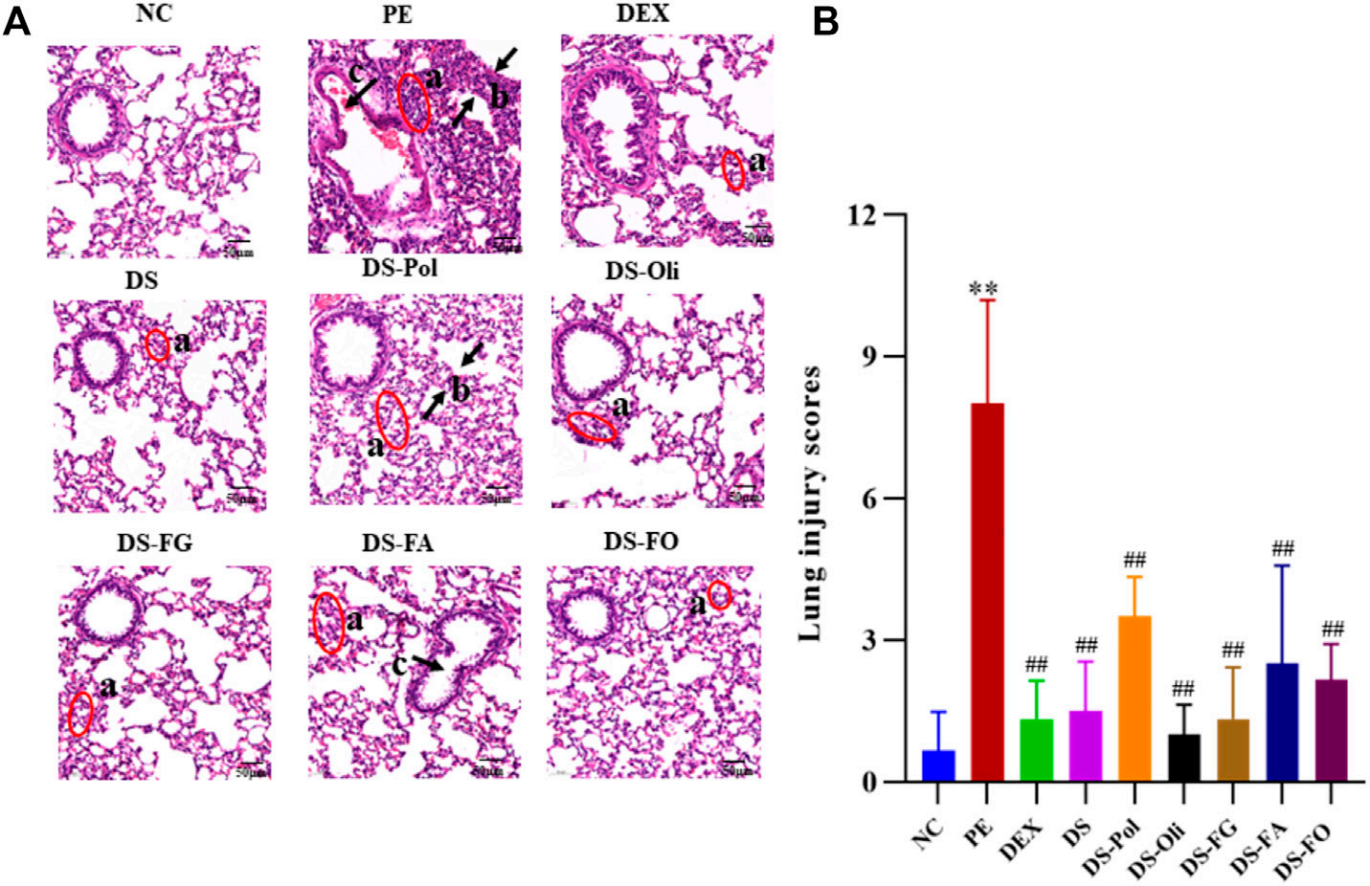
Effects of DS and its five fractions on lung histopathological change of PE model **(A)**. And their semiquantitative score of lung edema severity **(B)**. All data were expressed as mean ± SD, *n* = 3, **p < 0.01, vs. NC; ^##^ *p* < 0.01, vs. PE. The above lung sections chosen randomly from three left lung lobes sample of each group, respectively, were stained with hematoxylin and eosin (H&E, ×200). a, infiltration of inflammation; b, thickening of the alveolar wall; c, narrowing of airway lumina.

Pathology sections of lung tissue from all groups are shown in Figure 1A. In the NC group, the lung structure and alveolar space were clear and obvious morphologic damage was absent. In the PE model group, lung tissues showed severe infiltration of inflammatory cells, increased thickness of the alveolar wall, and narrowing of airway lumina. After treatment with DEX or DS, pathologic injury to the lung was attenuated markedly. Infiltrated inflammatory cells were present in the five DS-fraction groups, but the severity of pulmonary damage was lessened to different degrees. DS-Oli, DS-FG, and DS-FO fractions showed less damage to lung tissue than that of DS-Pol and DS-FA fractions. As seen in Figure 1B, the scoring of PE group was much higher than NC; compared with PE group, the scoring of all treatment groups was decreased in different degrees and there was significance between groups.

### 3.2 Validation of the LC-MS condition

**FIGURE 2:**
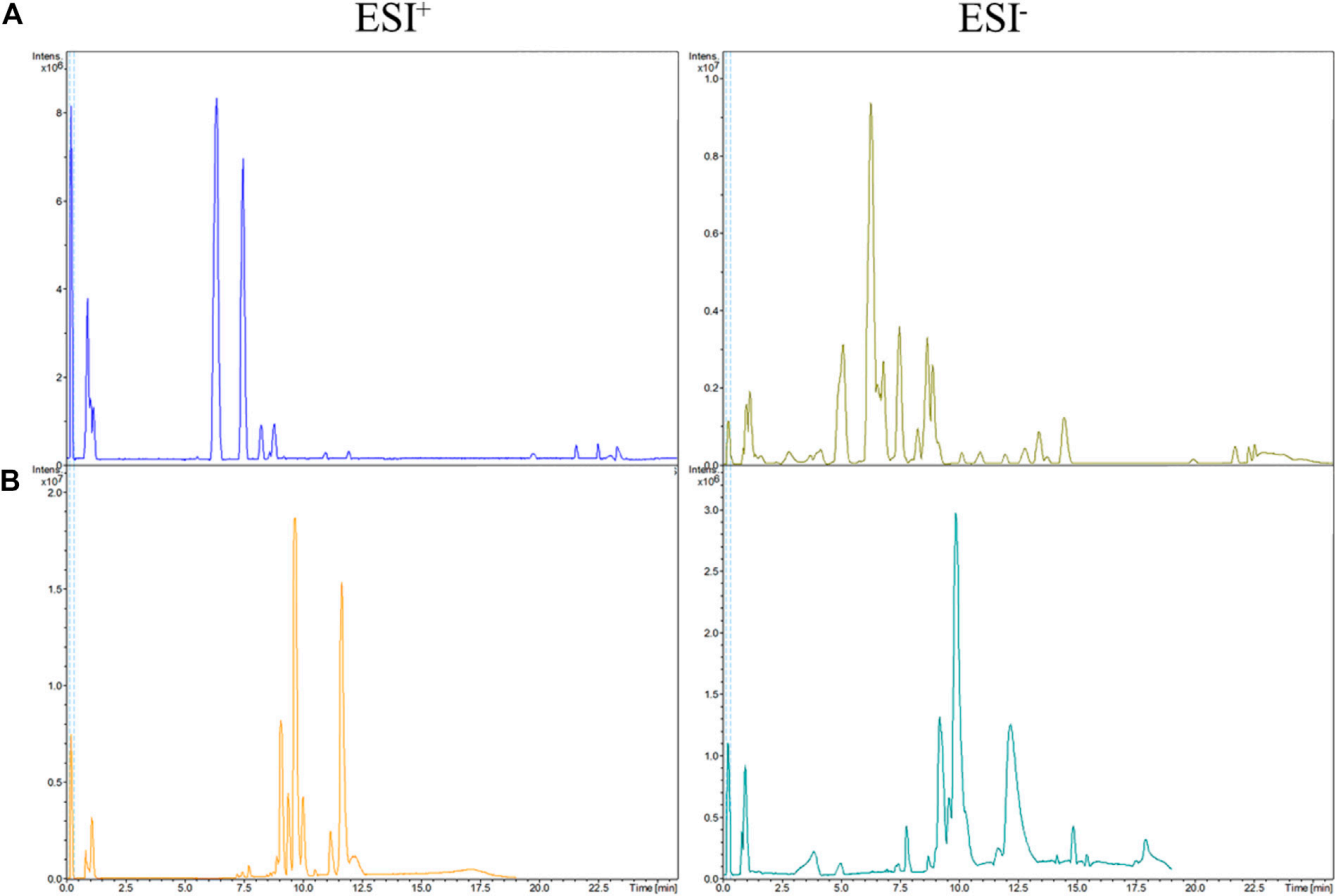
Base peak chromatograms in ESI^+^ and ESI^−^ modes. **(A)** urine QC, **(B)** serum QC.

To obtain excellent resolution of chromatographic peaks, separation parameters (column temperature, flow rate, injection volume) were optimized fully. The relative base peak QC chromatograms for urine and serum samples are exhibited in Figure 2.

**FIGURE 3:**
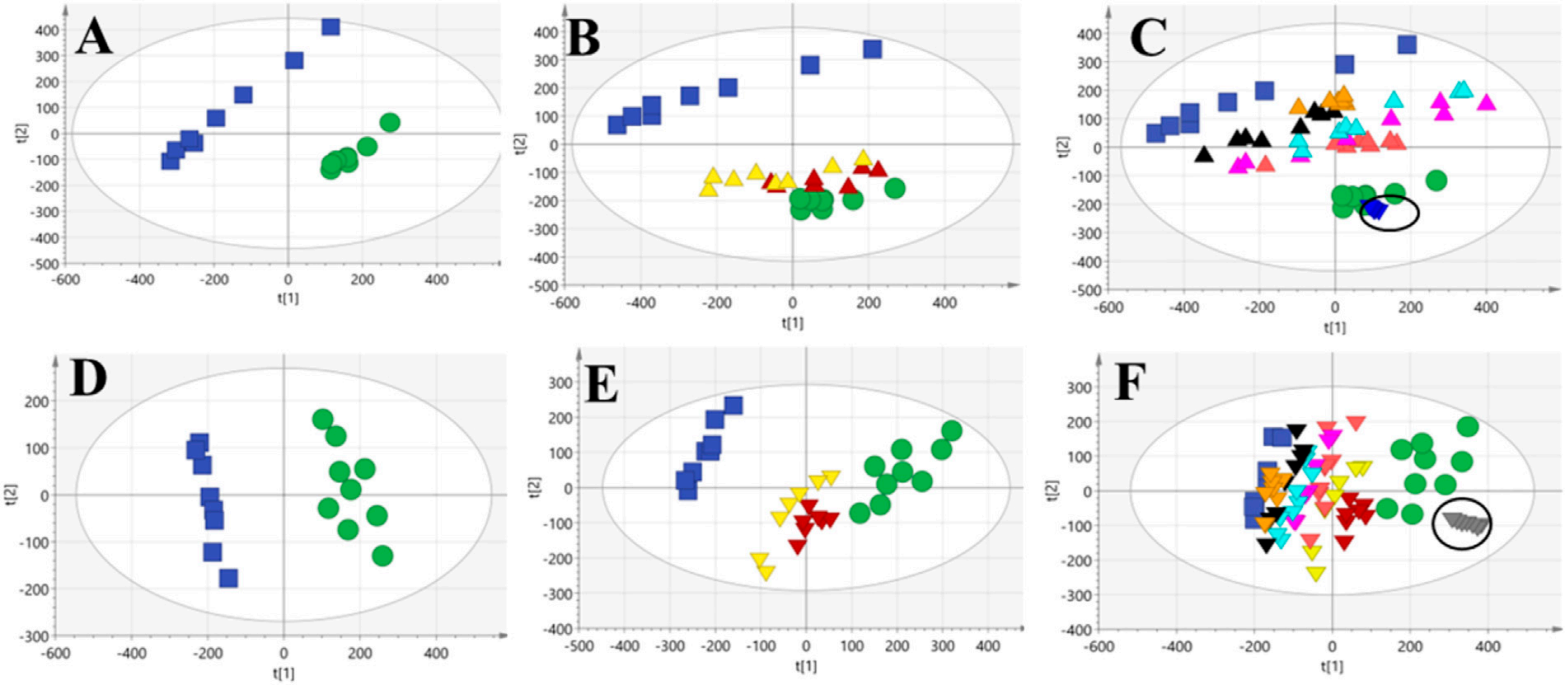
PCA score plots obtained from the control, M and treatment groups of urine and serum. NC and PE groups **(A,D)**; C, PE, DEX and DS groups **(B,E)**; NC, PE and six DS-treated groups **(C,F)**, suggesting the metabolic profiles variables among different groups. (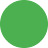
NC group, 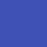
PE group, 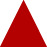
DEX group, 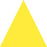
DS group, 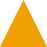
DS-Pol group, 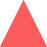
DS-Oli group, 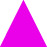
DS-FG group, 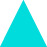
DS-FA group, 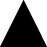
DS-FO group, 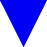
QC of urine, 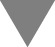
QC of serum). In serum samples, the inverted triangles with the same colors as urine samples represent the corresponding groups. NC, normal control; DS, *Descurainia sophia* seeds; DEX, Dexamethasone; DS-Pol, polysaccharides; DS-Oli, oligosaccharides; DS-FG, flavonoid glycosides; DS-FA, flavonoid aglycone; DS-FO, fat oil fraction.

To further assess the performance of our analytical system, PCA score plots of QC samples were obtained (Figures 3C, F). Six QC-sample points in PCA score plots aggregated together, which indicated the excellent repeatability and accuracy of our analytical system. Ten representative ions were chosen from urine and serum QC samples in positive and negative ion modes, respectively. The relative standard deviation (RSD) of the extracted peak areas and retention time were calculated (Table 1). RSD values were <30%, indicating the high reliability of our LC-MS method.

**TABLE 1:** RSDs of retention times and peak areas of ten potential biomarkers extracted from urine and serum of QCs.

	Adduct ion	m/z	Metabolites	RSDs of Rt (%)	RSDs of peak area (%)
1	M-H (urine)	124.0072	Taurine	0.67	18.44
2	M-H (urine)	173.0091	cis-Aconitic acid	0.54	23.32
3	M-H (urine)	117.0192	Succinic acid	0.44	24.21
4	M + H (urine)	206.0446	Xanthurenic acid	0.91	20.54
5	M + H (urine)	129.0658	L-Glutamine	0.54	9.39
6	M-H (serum)	103.0400	3-Hydroxybutyric acid	0.45	16.97
7	M-H (serum)	391.2853	Ursodeoxycholic acid	0.09	25.12
8	M + Na (serum)	140.0681	Betaine	0.72	13.19
9	M + H (serum)	300.2890	Sphingosine	0.07	18.85
10	M + H (serum)	166.0862	L-Phenylalanine	0.57	6.24

### 3.3 Metabolic profiles of urine and serum samples

PCA of urine and serum samples was carried out, respectively, to observe directly overall metabolic profile change of all groups after modeling and DS, DS-Pol, DS-Oli, DS-FG, DS-FA, and DS-FO administration. In urine samples, a complete separation was observed between the NC group and PE group (R^2^X = 0.684; Q^2^ = 0.54) (Figure 3A), which indicated that modeling had disturbed the endogenous metabolites of rats significantly and resulted in deviation of PE-model rats from a healthy state. Besides, in our previous pharmacological experiment, compared with NC group, a significantly increased lung wet/dry weight ratio, lung indices, lung water content, pleural effusion accumulation, amount of leukocyte extravasation, as well as reduced interleukin (IL)-4 and immunoglobulin-E (IgE) levels in bronchoalveolar lavage fluid could be observed after modeling with carrageenan (Wang, M. M. et al., 2022). Coupled with remarkable separation of the metabolic profile between PE and NC groups suggested that the PE model had been established successfully. The metabolic profile of DEX and DS groups was close to that of normal rats but very distant to that of the PE group (R^2^X = 0.71, Q^2^ = 0.587) (Figure 3B), which suggested that the metabolic perturbation of PE-model rats had been reversed to the normal state by administration of DEX or DS. We wished to investigate the contribution of the five fractions to the effects of DS. Hence, NC, PE, DS, and the five DS-fraction groups were analyzed (R^2^X = 0.768, Q^2^ = 0.646) (Figure 3C). The distribution of effects of the five fractions groups was between the NC group and model group. Therefore, the five fractions could ameliorate the disordered endogenous metabolites in some way, and their combined effects constituted the entire efficacy of DS. However, the degree of improvement varied considerably. DS-Oli, DS-FG, DS-FA, and DS-FO groups (especially DS-Oli) were closer to the NC group compared with the DS-Pol group, which suggested that DS-Oli might be a major contributor to DS for recovering the irregular metabolic profile of PE. The DS-Pol group deviated from the NC group and nearly clustered together with the model group. This finding revealed that the DS-Pol fraction might exert weaker efficacy than that of the other four fractions of DS on PE, which was similar to the histology result. Coincidentally, the results of PCA of serum were highly consistent with those of urine [R^2^X = 0.517, Q^2^ = 0.33 (Figure 3D), R^2^X = 0.595, Q^2^ = 0.494 (Figure 3E), R^2^X = 0.651, Q^2^ = 0.551 (Figure 3F)].

### 3.4 Potential biomarkers

We wished to further investigate differential metabolites between PE vs. NC, DS vs. PE, DS-Pol vs. PE, DS-Oli vs. PE, DS-FG vs. PE, DS-FA vs. PE, and DS-FO vs. PE and determine the role of DS, DS-Pol, DS-Oli, DS-FG, DS-FA and DS-FO in treating PE. Hence, based on PCA, seven couple of OPLS-DA score scatter plots were constructed in urine and serum samples, respectively. As shown in OPLS-DA score plots (Supplementary Material S1), the model group separated completely from NC, DS, DS-Pol, DS-Oli, DS-FG, DS-FA, and DS-FO groups, and all values of R^2^X, R^2^Y, and Q^2^ are presented in Supplementary Material S2. The corresponding S-plots were generated to obtain altered metabolites that played a key part in discriminating these groups. All OPLS-DA models had good validity through 200 permutation tests.

**FIGURE 4:**
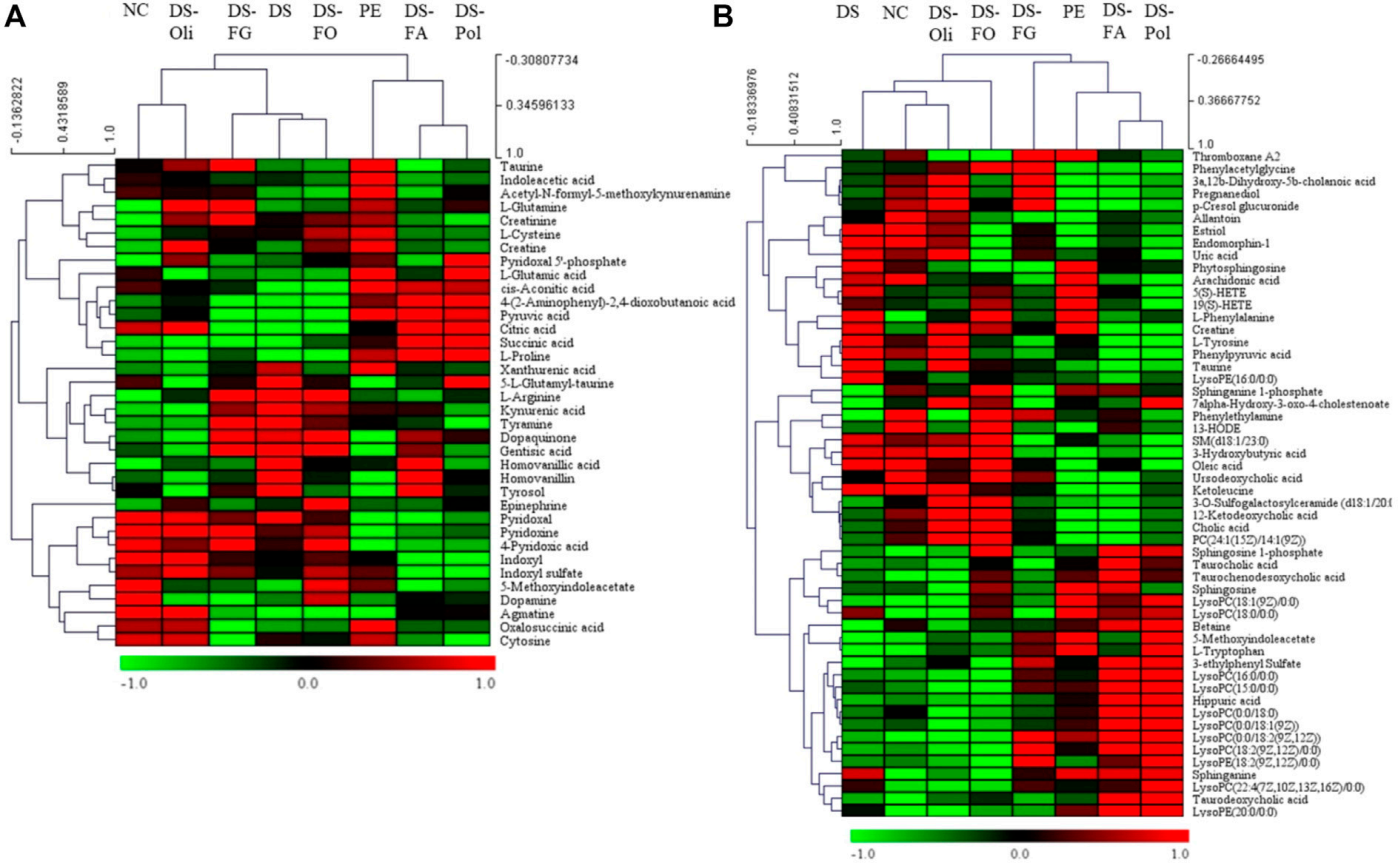
Heat maps and hierarchical clustering analysis (HCA) of potential biomarkers in urine **(A)** and serum samples **(B)**. The color from red to blue referred to higher and lower average content changes of the potential biomarkers, respectively. The rows represent different metabolites, and the columns represent different groups.

According to the Student’s t-test (*p* < 0.05) and VIP value (>3), the significantly changed biomarkers that differentiated the model group from the NC group and six DS-treatment groups were filtered out in urine and serum samples. A total of thirty-six potential biomarkers (urine) and 54 biomarkers (serum) derived from PE vs. NC, DS vs. PE, DS-Pol vs. PE, DS-Oli vs. PE, DS-FG vs. PE, DS-FA vs. PE, and DS-FO vs. PE were identified (Supplementary Materials S3, S4). Heatmaps were created and HCA were undertaken to visualize the mean change in content of biomarkers in all groups. DS-Pol, DS-FA, and model groups were clustered into one category, whereas NC, DS, DS-Oli, DS-FG, and DS-FO groups were clustered into another category [Figure 4A (urine), Figure 4B (serum)]. These results revealed that DS-Pol and DS-FA fractions might have a weaker effect against PE than that proffered by DS-Oli, DS-FG, or DS-FO fractions.

### 3.5 Correlation between the metabolic pathways of disturbed biomarkers

**FIGURE 5:**
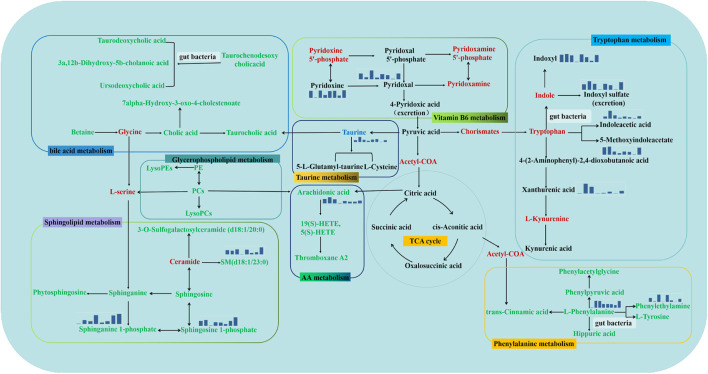
Metabolic pathway analysis, reflecting the inner relationship among potential biomarkers intervened by DS, DS-Pol, DS-Oli, DS-FG, DS-FA, DS-FO treatment. It was marked red as absent metabolites, metabolites labeled in black were from urine, labeled in green were from serum. Mini bar chart represents average content of main biomarkers in NC, PE, DS, DS-Pol, DS-Oli, DS-FG, DS-FA, DS-FO groups.

For urine samples, 36 biomarkers were imported into MetaboAnalyst and MBRole 2.0 for analyses of enrichment of metabolic pathways. “Vitamin B6 metabolism”, “taurine metabolism”, and “tryptophan metabolism” were affected by modeling and administration of DS, DS-Pol, DS-Oli, DS-FG, DS-FA, or DS-FO. Five perturbed pathways were enriched in serum: “phenylalanine metabolism”, “arachidonic acid metabolism”, “sphingolipid metabolism”, “bile acid metabolism”, and “glycerophospholipid metabolism”. These pathways mainly associated with inflammation, edema clearance etc. All of pathways were closely interconnected with each other in the metabolic network (Figure 5).

## 4 Discussion

### 4.1 Pathways involved inflammation

#### 4.1.1 Vitamin B6 metabolism

In urine, vitamin B6 metabolism was one of significantly co-regulated metabolic pathways in all six DS-treatment groups. Vitamin B6 has a crucial role in antioxidant and inflammatory mechanisms because it inhibits lipid peroxidation (Chiang et al., 2005). Vitamin B6 is a general term for a class of bioactive substances: pyridoxine, pyridoxamine, pyridoxal, and their phosphorylated forms (Brown et al., 2022). It has been reported that vitamin B6 deficiency can reduce its antioxidant capacity or promote inflammatory reactions directly (Lee et al., 2018). Further study observed that the pyridoxal level was inversely associated with the degree of inflammatory response and oxidative stress, and that a low pyridoxine level could enhance inflammation (Liu et al., 2021). Compatible with those observations, the content of pyridoxal and pyridoxine in the urine of the model group decreased, which could further aggravate inflammatory damage to the lung. In DS-Oli, DS-FG, and DS-FO groups, the level of pyridoxal and pyridoxine was increased to inhibit the degree of the inflammatory response in PE treatment. Therefore, we speculate that DS-Oli, DS-FG, or DS-FO may regulate (at least in part) the synthesis of pyridoxal and pyridoxine in the vitamin B6 pathway to attenuate the inflammatory response and promote antioxidant abilities, thereby improving the lung injury observed in PE. However, the regulatory effect of DS-Pol and DS-FA was not obvious.

#### 4.1.2 Taurine metabolism

Taurine is a prevalent intracellular free amino acid. It possesses four main biological functions: i) taurine acts as an physiological antioxidant to reduce oxidative stress (Baliou et al., 2021); ii) cytoprotection in acute inflammation and anti-apoptosis effects (Lambert et al., 2015). iii) regulation of osmotic pressure (Chesney et al., 2010); iv) maintenance of calcium homeostasis and membrane stabilization (Han et al., 2000). Taurine has been demonstrated to inhibit the lung damage caused by oxidative stress by enhancing the activity of antioxidWWant enzymes in the lung (Schuller-Levis et al., 1994; Abdih et al., 2000; Zhao et al., 2018). Chen and colleagues discovered that taurine can attenuate sepsis-induced lung injury through anti-inflammatory actions and antioxidative stress, reducing overexpression of tumor necrosis factor (TNF)-α and IL-1β and neutrophil infiltration (Chen, J. et al., 2021). Early studies also showed that abnormalities of the taurine metabolism of occurred in viral pneumonia and lipopolysaccharide-induced pulmonary injury (Chen, F. et al., 2022). Hence, taurine is regarded as a potential agent for lung injury under various conditions. In our study, taurine excretion was increased considerably in the urine of model-group rats coWWWmpared with that in NC-group rats, which may have led to relative insufficiency *in vivo* to protect the lung against inflammation and oxidative damage. After intervention with DS or its fractions, the taurine level in urine decreased, indicating that DS, DS-Pol, DS-Oli, DS-FG, DS-FA, or DS-FO might promote taurine accumulation of taurine *in vivo* to enable distribution to lung tissue to alleviate inflammation and oxidative stress. Consistently, a decrease in the serum level of taurine in the model group and an increase in treatment groups were observed (Supplementary Material S4), further bolstering the hypothesis made above.

On the other hand, taurine has been shown to be renoprotective in various models (Han and Chesney, 2012). Also, taurine facilitates natriuresis and diuresis through mediating renal osmoregulatory activity (Schaffer et al., 2000). TCM and pharmacology studies have shown pulmonary function to be linked closely to kidney function, taking part together in modulating fluid–water balance (Malek et al., 2018). Lung dysfunction would affect kidney function, resulting in the symptoms of oliguria and edema (Karajala et al., 2009; Domenech et al., 2017). In addition, the greatly increased urine creatinine level in the model group combined with reduced urine output denoted renal injury. Therefore, DS, DS-Pol, DS-Oli, DS-FG, DS-FA, and DS-FO could also adjust taurine content to improve renal function, thereby increasing urine volume to reduce edema and cardiac load indirectly in PE. This hypothesis explains (at least in part) the diuretic effect of DS from a metabolic viewpoint as well.

#### 4.1.3 Tryptophan metabolism

Tryptophan is an essential amino acid found in mammals. Tryptophan metabolism disorder is closely associated with lung injury by mediating immunity homeostasis, gut microbiota and inflammatory response (Platten et al., 2019). Tryptophan is metabolized mainly by a rate-limiting enzyme, indoleamine 2,3-dioxygenase (IDO) through the kynurenine pathway (KP) (Badawy, 2017). IDO can be driven by the gut microbiota and proinflammatory cytokines (e.g., interferon-γ, TNF-α, prostaglandin-E2 (Li et al., 2020; Liu et al., 2021; Zhou, N. et al., 2018). In the present study, levels of tryptophan catabolites through the KP (e.g., kynurenic acid, xanthurenic acid) were increased in the model group, suggesting upregulated IDO activity, microbial dysbiosis and release of proinflammatory factors in PE. Disorders in the gut microbiota have been shown to aggravate lung inflammation through the lung–gut axis (Budden et al., 2017; Zhou, X. and Liao, 2021). And, the disturbance of KP could further lead to oxidate stress and induce lung apoptosis (Li et al., 2020). In addition, tryptophan can also be converted directly into indole and its derivatives by the action of gut microbiota, including indoleacrylic acid, indoleacetic acid (IAA), and indolepropionic acid. These derivatives can act as ligands of aryl hydrocarbon receptors to maintain homeostasis of the immune system (Agus et al., 2018; Comai et al., 2020). Here, levels of these tryptophan catabolites returned to normal after treatment with DS, DS-Pol, DS-Oli, DS-FG, DS-FA, or DS-FO. Hence, DS and its five fractions might maintain gut-microbiota balance and pulmonary immune homeostasis by regulating IDO activity in tryptophan metabolism, thus ameliorating lung injury.

#### 4.1.4 Arachidonic acid (AA) metabolism

AA is an essential fatty acid. It can be metabolized into different bioactive substances by cyclooxygenase, lipoxygenase, or cytochrome P450 enzymes (P450) and regulate complicated inflammatory responses (Sonnweber et al., 2018). Thromboxanes, produced by cyclooxygenase, can mediate platelet aggregation. 5-Hydroxyeicosatetraenoic acid [5(S)-HETE] is a 5-lipoxygenase product, affects neutrophil recruitment, epithelial-barrier function, and vascular permeability. 19(S)-HETE is a metabolite of the P450 pathway, and can regulate the constriction and dilation of blood vessels (Wang, T. et al., 2019). MA of serum samples revealed an increase in levels of AA and its three derivatives in model-group rats. This result indicated that AA metabolism was strengthened, which was consistent with severe infiltration of inflammatory cells and histology findings (Figure 1). Also, administration of DS, DS-Pol, DS-Oli, DS-FG, DS-FA, or DS-FO could decrease levels of these metabolites to different degrees (Supplementary Material S3). Thereupon, pulmonary inflammation was relieved, which illustrated that the five fractions from DS might exert a synergistic effect together with inhibiting inflammatory damage to the lung through mediating AA metabolism.

### 4.2 Pathways involved edema clearance

#### 4.2.1 Phenylalanine metabolism

In serum, phenylalanine is an essential amino acid. However, phenylalanine takes part in several pathophysiologic processes if its concentration is increased rapidly (Tan et al., 2020). A significantly increased phenylalanine level was detected in the serum of PE-model rats compared with that in NC-group rats, indicating that phenylalanine metabolism may have been restrained. Furthermore, levels of the downstream metabolites of phenylalanine, such as phenylacetylglycine and phenylpyruvic acid, were reduced, which also suggested that phenylalanine was not metabolized completely. Inflammation can decrease the activity of phenylalanine hydroxylase and then impair its conversion to L-Tyrosine (Okusaga et al., 2014), thereby resulting in phenylalanine accumulation and reduced levels of L-Tyrosine, data which are consistent with our results (Supplementary Material S4). An early study suggested that an increase in the phenylalanine level in blood may indirectly inhibit the activity of Na^+^/K^+^ATPase and alter its functions (Schulpis et al., 2002). Scholars have shown Na^+^/K^+^ATPase to be a ubiquitous transmembrane protein expressed on the basolateral surface of most mammalian epithelial cells. Na^+^/K^+^ATPase participates in the active transport of Na^+^ across the alveolar epithelium and regulates epithelial-barrier function (Vadasz et al., 2007), which has a key role in the resolution of lung edema. Therefore, downregulation of Na^+^/K^+^ATPase activity can decrease active transport of Na^+^, resulting in the reduced ability of the lung to clear edema (Wang, Q. et al., 2013). After administration of DS or its fractions, levels of phenylalanine and relevant metabolites tended to return to normal. Thus, DS, DS-Oli, DS-FG, or DS-FO could activate Na^+^/K^+^ATPase to promote transport of alveolar fluid by promoting phenylalanine metabolism and thereby mitigating lung-edema fluid in the alveolar cavity. However, the degree of regulation of these metabolites varied; DS-FA and DS-Pol fractions did not exert this effect.

#### 4.2.2 Sphingolipid metabolism

Sphingolipids are a class of essential lipids found in cell membranes. They can regulate the immunity, proliferation, inflammation signaling, migration, adhesion, and cytoskeletal rearrangement of cells (Hannun and Obeid, 2018). Ceramide and sphingosine-1-phosphate (S1P) are major mediators involved in acute lung injury (Brinkmann and Baumruker, 2006; Ebenezer et al., 2016). Ceramide can be converted from sphingomyelin by sphingomyelinase (aSMase), whereas S1P is produced from ceramide via sphingosine-kinases (Lin et al., 2011). Serum S1P plays a key part in maintaining vascular homeostasis, which is crucial for the pathophysiologic processes of pulmonary diseases (Thuy et al., 2014). S1P enhances the barrier function of the lung capillary endothelium through S1P receptor1-mediated rearrangement of action microfilaments and maintains vascular integrity by tightening intercellular adhesion, thereby decreasing vascular permeability and alveolar edema in lung inflammation (Obinata and Hla, 2019). Ceramide exerts opposite effects: it increases vascular leakage and the apoptosis of endothelial cells to induce lung edema. Besides, the rate-limiting enzyme aSMase can be activated by proinflammatory factors and platelet-activating factor to increase ceramide synthesis in response to lung injury (Yagci et al., 2019). In the present study, ceramide might increase in model group due to the down-regulation of its downstream products including SM(d18:1/23:0) and 3-O-Sulfogalactosylceramide (d18:1/20:0). The S1P level was also increased in the model group. Therefore, levels of ceramide and S1P were increased in the serum of PE-model rats (Supplementary Material S4). The reason for the increase in concentration of ceramide and S1P might be that S1P protects against ceramide-caused tissue injury under inflammatory stimuli, which represents self-defense *in vivo* (von Bismarck et al., 2012). The S1P content in the DS-Oli group and DS-FG group tended to be normal, and levels of the relevant metabolites in this pathway tended to return to normal in the DS group. Also, the dynamic equilibrium between ceramide and S1P is crucial to maintain homeostasis of alveolar functions (Petrache and Berdyshev, 2016). Hence, treatment with DS, DS-Oli, or DS-FG could improve vascular leakage and lung-tissue damage, and maintain the integrity of the alveolar capillary membrane by altering the dynamic balance of ceramide and S1P in sphingolipid metabolism.

#### 4.2.3 Bile acid metabolism

Ursodeoxycholic acid (UDCA) is a beneficial metabolite produced by intestinal bacteria. It has hepatoprotective effects through anti-inflammatory, anti-apoptotic, and immunomodulatory activities (Kim, D. J. et al., 2018; Tan et al., 2020). Recent studies have suggested that UDCA has a good effect against pulmonary diseases. UDCA has been shown to relieve airway inflammation through suppression of dendritic-cell function, inhibition of airway remodeling, apoptosis of airway epithelial cells, as well as a T-helper-type-2 immune response in asthma (Isik et al., 2017). UDCA can stimulate alveolar-fluid clearance through mediating related signaling pathway in lipopolysaccharide-induced pulmonary edema, which is important for lung gas exchange and edema elimination (Willart et al., 2012; Niu et al., 2019). In the present study, the serum UDCA level was reduced after modeling in comparison with the NC group and returned to a normal level after treatment with DS, DS-Pol, DS-Oli, DS-FG, or DS-FO. Thus, DS and its fractions could enhance pulmonary alveolar-fluid clearance to maintain normal gas exchange, repair airway epithelial cells, and improve airway inflammation by increasing the serum UDCA level, which could be one cause of attenuated dyspnea. However, the DS-FA fraction had a slightly weaker effect.

### 4.3 Other pathways involved pulmonary function

#### 4.3.1 Glycerophospholipid metabolism

Phosphatidylcholines (PCs) are parts of cell membranes and are lung surfactants, and maintain normal respiratory function (Akella and Deshpande, 2013). Lysophosphatidylcholines (LysoPCs) are bioactive lipids synthesized from the hydrolysis or oxidation of PCs via phospholipase A2 and released into blood under physiological conditions (Lindahl et al., 1988; Knuplez and Marsche, 2020). In general, lysoPCs are considered to be potent proinflammatory mediators and biomarkers of lipid peroxidation *in vivo* (Yuan, A. et al., 2017). LysoPCs release can lead to damage to tissues and cells through upregulation of adhesion molecules, as well as increased endothelial permeability and apoptosis through platelet-activating factor receptors (Hahnefeld et al., 2021). The level of lysoPCs is increased in certain inflammatory states. Reduced levels of PCs and increased levels of lysoPCs were observed in the model group (Figure 5B), indicating that the conversion from PCs to lysoPCs was enhanced in the model group. The content of PCs and lysoPCs was reversed and approached that of healthy rats in DS, DS-Oli, DS-FG, and DS-FO groups. Hence, regulation of levels of lysoPCs and PCs in glycerophospholipid metabolism contributed to recovery of respiratory function, but DS-Pol and DS-FA fractions did not have curative efficacy.

### 4.4 Summary

All five fractions of DS could alleviate lung inflammatory injury, maintain homeostasis of the immune system and intestinal microbiota, promote kidney function, and exert a diuretic effect by regulating the metabolism of vitamin B6, taurine, tryptophan and AA. Besides, DS-Oli, DS-FG and DS-FO fractions could contribute to accelerating alveolar-fluid transport, repair the endothelial barrier, and decrease vascular leakage by regulating the metabolism of phenylalanine, sphingolipids, bile acids metabolism, and glycerophospholipids. Therefore, DS-Oli, DS-FG, and DS-FO coupled with DS-Pol and DS-FA had a synergistic role in relieving the pathologic damage and symptoms of PE, thereby constituting the overall efficacy of DS.

## 5 Conclusion

Our findings showed five DS fractions ameliorated lung injury by regulating perturbed metabolic pathways involved inflammatory in PE. However, DS-Oli, DS-FG, and DS-FO exerted higher efficacy compared with DS-Pol and DS-FA in terms of edema liquid clearance or reabsorption. Combining the comprehensive results of lung histology, metabolic-pathway regulation in urine and serum samples, heatmaps and HCA suggested that DS-Oli, DS-FG, or DS-FO could be used as an alternative to DS for PE treatment. Our integrated MA strategy provides a powerful tool to understand the holistic and therapeutic effect of a TCM and evaluate the role of its fractions.

## Data Availability

The original contributions presented in the study are included in the article/Supplementary Materials, further inquiries can be directed to the corresponding authors.

## Abbreviations

DS, Descurainia sophia seeds; TCM, traditional Chinese medicine; PE, pulmonary edema; MA, metabolomics analysis; DEX, Dexamethasone; UHPLC-Q/TOF-MS, ultra-high-performance liquid chromatography-quadrupole time-of-flight mass spectrometry; PCA, principal component analysis; OPLS-DA, orthogonal partial least squares-discriminant analysis; DS-Pol, polysaccharides; DS-Oli, oligosaccharides; DS-FG, flavonoid glycosides; DS-FA, flavonoid aglycone; DS-FO, fat oil fractio; ESI, eletrospray ionization source; ELSD, evaporative light scattering detector; PDA, photodiode array detector; UA, ultraviolet detector; RSD, relative standard deviations; VIP, variable importance of project; 5(S)-HETE, 5(S)-hydroxyeicosatetraenoic acid.
