# Supplementary material for: Protective effects of Descurainia sophia seeds extract and its fractions on pulmonary edema by untargeted urine and serum metabolomics strategy
Source: Front Pharmacol. 2023 Feb 14;14:1080962. doi: 10.3389/fphar.2023.1080962 (PMC9971919; doi:10.3389/fphar.2023.1080962)
Supplement: Supplementary file 1 [file DataSheet4.docx]

**Supplementary Material 4**

**TABLE S2** Identified biomarkers related to PE/DS-Pol/DS-Oli/ DS-FG/DS-FA/DS-FO in serum.

| **NO.** | **Detected m/z** | **Rt**  **(min)** | **Metabolites** | **Formula** | **Adduct Ion** | **PE**  ***vs.* NC^a^** | **DS^b^**  ***vs.*PE** | **DS-Pol^b^**  ***vs.* PE** | **DS-Oli^b^**  ***vs.* PE** | **DS-FG^b^**  ***vs.* PE** | **DS-FA^b^**  ***vs.* PE** | **DS-FO^b^**  ***vs.* PE** |
| --- | --- | --- | --- | --- | --- | --- | --- | --- | --- | --- | --- | --- |
| 1 | 166.0862 | 1.1 | L-Phenylalanine | C_9_H_11_NO_2_ | M+H | ↑**, √ | ↓, √ | ↓**, √ | ↓**, √ | ↓**, √ | ↓, √ | ↓*, √ |
| 2 | 182.0808 | 1.0 | L-Tyrosine | C_9_H_11_NO_3_ | M+H | ↓, √ | ↑*, √ | ↑*, √ | ↓, √ | ↓* | ↓ | ↓* |
| 3 | 194.0805 | 4.1 | Phenylacetylglycine | C_10_H_11_NO_3_ | M+H | ↓, √ | ↑, √ | ↑ | ↑ | ↓, √ | ↑ | ↓ |
| 4 | 165.0543 | 1.0 | Phenylpyruvic acid | C_9_H_8_O_3_ | M+H | ↓, √ | ↑**, √ | ↑*, √ | ↓, √ | ↓*, √ | ↑, √ | ↓*, √ |
| 5 | 180.0651 | 2.9 | Hippuric acid | C_9_H_9_NO_3_ | M+H | ↑, √ | ↓ | ↓*, √ | ↓ | ↑ | ↓ | ↑** |
| 6 | 122.0963 | 13.8 | Phenylethylamine | C_8_H_11_N | M+H | ↓*, √ | ↓, √ | ↓, √ | ↑, √ | ↑, √ | ↑, √ | ↓, √ |
| 7 | 401.3447 | 8.9 | SM(d18:1/23:0) | C_46_H_93_N_2_O_6_P | M+2H | ↓, √ | ↑, √ | ↑, √ | ↓, √ | ↓, √ | ↑*, √ | ↓*, √ |
| 8 | 818.5853 | 7.7 | 3-O-Sulfogalactosylceramide (d18:1/20:0) | C_44_H_85_NO_11_S | M+H-H2O | ↓, √ | ↑ | ↑** | ↑, √ | ↓, √ | ↑ | ↑, √ |
| 9 | 300.2890 | 8.1 | Sphingosine | C_18_H_37_NO_2_ | M+H | ↑**, √ | ↓** | ↓**, √ | ↓**, √ | ↓ | ↓ | ↓** |
| 10 | 378.2416 | 8.4 | Sphingosine 1-phosphate | C_18_H_38_NO_5_P | M-H | ↑, √ | ↓ | ↓, √ | ↓ | ↑* | ↑**, √ | ↑*, √ |
| 11 | 380.2570 | 8.7 | Sphinganine 1-phosphate | C_18_H_40_NO_5_P | M-H | ↑, √ | ↓*, √ | ↓ | ↓ | ↓, √ | ↑ | ↓ |
| 12 | 318.2998 | 7.5 | Phytosphingosine | C_18_H_39_NO_3_ | M+H | ↑*, √ | ↓, √ | ↓**, √ | ↓**, √ | ↓*, √ | ↓** | ↓*, √ |
| 13 | 302.3047 | 8.2 | Sphinganine | C_18_H_39_NO_2_ | M+H | ↑*, √ | ↓, √ | ↓, √ | ↓, √ | ↓, √ | ↓, √ | ↑, √ |
| 14 | 305.2466 | 10.4 | Arachidonic acid | C_20_H_32_O_2_ | M+H | ↑, √ | ↓ | ↓*, √ | ↓** | ↓**, √ | ↓, √ | ↓** |
| 15 | 338.3416 | 12.2 | Thromboxane A2 | C_20_H_40_O | M+ACN+H | ↑, √ | ↓, √ | ↓*, √ | ↓, √ | ↓, √ | ↓, √ | ↓, √ |
| 16 | 303.2319 | 10.0 | 5(S)-HETE | C_20_H_32_O_3_ | M+H-H2O | ↑, √ | ↓, √ | ↓, √ | ↓, √ | ↓, √ | ↓, √ | ↓**, √ |
| 17 | 319.2282 | 10.1 | 19(S)-HETE | C_20_H_32_O_3_ | M-H | ↑, √ | ↓, √ | ↓, √ | ↓, √ | ↓, √ | ↓, √ | ↓**, √ |
| 18 | 124.0073 | 1.0 | Taurine | C_2_H_7_NO_3_S | M-H | ↓, √ | ↑**, √ | ↑** | ↑ | ↓ | ↑ | ↓ |
| 19 | 373.2731 | 7.3 | Cholic acid | C_24_H_40_O_5_ | M+H-2H2O | ↓ | ↑, √ | ↑**, √ | ↑, √ | ↓, √ | ↑, √ | ↑, √ |
| 20 | 496.2733 | 7.2 | Taurocholic acid | C_26_H_45_NO_7_S | M-H20-H | ↑, √ | ↓, √ | ↓ | ↓, √ | ↑*, √ | ↓, √ | ↑, √ |
| 21 | 431.3148 | 9.4 | 7alpha-Hydroxy-3-oxo-4-cholestenoate | C_27_H_42_O_4_ | M+H | ↑, √ | ↓ | ↑, √ | ↓ | ↓ | ↑ | ↑ |
| 22 | 498.2888 | 7.1 | Taurochenodesoxycholic acid | C_26_H_45_NO_6_S | M-H | ↑, √ | ↓ | ↓ | ↓, √ | ↑ | ↓, √ | ↑, √ |
| 23 | 391.2853 | 8.9 | Ursodeoxycholic acid | C_24_H_40_O_4_ | M-H | ↓**, √ | ↑, √ | ↑ | ↑, √ | ↓, √ | ↑**, √ | ↑ |
| 24 | 405.2645 | 7.5 | 3a,12b-Dihydroxy-5b-cholanoic acid | C_24_H_40_O_4_ | M-H | ↓, √ | ↑, √ | ↑* | ↑*, √ | ↓, √ | ↑ | ↑, √ |
| 25 | 498.2892 | 8.0 | Taurodeoxycholic acid | C_26_H_45_NO_6_S | M-H | ↑, √ | ↓, √ | ↓, √ | ↓, √ | ↑ | ↑, √ | ↑**, √ |
| 26 | 355.2628 | 7.7 | 12-Ketodeoxycholic acid | C_24_H_38_O_4_ | M+H-2H2O | ↓, √ | ↑, √ | ↑**, √ | ↑, √ | ↓ | ↑, √ | ↑, √ |
| 27 | 140.0681 | 0.9 | Betaine | C_5_H_11_NO_2_ | M+Na | ↑, √ | ↓**, √ | ↓**, √ | ↓ | ↑ | ↓, √ | ↑** |
| 28 | 389.2679 | 7.4 | Pregnanediol | C_21_H_36_O_2_ | M+H+HCOONa | ↓, √ | ↑ | ↑** | ↑, √ | ↓, √ | ↑ | ↑, √ |
| 29 | 311.1617 | 11.2 | Estriol | C_18_H_24_O_3_ | M+Na | ↓**, √ | ↑**, √ | ↑** | ↑**, √ | ↑**, √ | ↑, √ | ↑, √ |
| 30 | 129.0556 | 2.6 | Ketoleucine | C_6_H_10_O_3_ | M-H | ↓ | ↑, √ | ↑ | ↑, √ | ↓ | ↑ | ↓ |
| 31 | 279.2312 | 13.1 | 13-HODE | C_18_H_32_O_3_ | M+H-H2O | ↓**, √ | ↑ | ↑, √ | ↑ | ↑, √ | ↑**, √ | ↑, √ |
| 32 | 103.0400 | 1.1 | 3-Hydroxybutyric acid | C_4_H_8_O_3_ | M-H | ↓**, √ | ↑**, √ | ↑** | ↑, √ | ↑, √ | ↑**, √ | ↓, √ |
| 33 | 132.0766 | 0.9 | Creatine | C_4_H_9_N_3_O_2_ | M+H | ↑**, √ | ↓, √ | ↓, √ | ↓*, √ | ↓**, √ | ↓, √ | ↓**, √ |
| 34 | 159.0508 | 1.0 | Allantoin | C_4_H_6_N_4_O_3_ | M+H | ↓**, √ | ↑, √ | ↑, √ | ↑, √ | ↑, √ | ↑, √ | ↑, √ |
| 35 | 188.0703 | 1.1 | 5-Methoxyindoleacetate | C_11_H_11_NO_3_ | M+H-H2O | ↑**, √ | ↓**, √ | ↓** | ↓**, √ | ↓**, √ | ↓**, √ | ↑, √ |
| 36 | 205.0971 | 1.1 | L-Tryptophan | C_11_H_12_N_2_O_2_ | M+H | ↑**, √ | ↓**, √ | ↓**, √ | ↓**, √ | ↓**, √ | ↓**, √ | ↑, √ |
| 37 | 283.2624 | 8.4 | Oleic acid | C_18_H_34_O_2_ | M+H | ↓**, √ | ↑** | ↑, √ | ↑, √ | ↑ | ↑** | ↓**, √ |
| 38 | 306.1510 | 9.6 | Endomorphin-1 | C_34_H_38_N_6_O_5_ | M+2H | ↓**, √ | ↑**, √ | ↑** | ↑**, √ | ↑**, √ | ↑**, √ | ↑ |
| 39 | 283.0819 | 4.9 | p-Cresol glucuronide | C_13_H_16_O_7_ | M-H | ↓ | ↑ | ↑* | ↑*, √ | ↓, √ | ↑ | ↓ |
| 40 | 167.0209 | 1.0 | Uric acid | C_5_H_4_N_4_O_3_ | M-H | ↓, √ | ↑ | ↑**, √ | ↑, √ | ↑ | ↓ | ↓ |
| 41 | 201.0227 | 6.3 | 3-ethylphenyl Sulfate | C_8_H_10_O_4_S | M-H | ↑ | ↓*, √ | ↓, √ | ↑, √ | ↑*, √ | ↓**, √ | ↑*, √ |
| 42 | 520.3400 | 9.1 | LysoPC(0:0/18:2(9Z,12Z)) | C_26_H_50_NO_7_P | M+H | ↑**, √ | ↓*, √ | ↓**, √ | ↓, √ | ↑, √ | ↓*, √ | ↑, √ |
| 43 | 544.3370 | 10.0 | LysoPC(18:1(9Z)/0:0) | C_26_H_52_NO_7_P | M+Na | ↑**, √ | ↓**, √ | ↓**, √ | ↓**, √ | ↓**, √ | ↓**, √ | ↓, √ |
| 44 | 524.3712 | 11.7 | LysoPC(18:0/0:0) | C_26_H_54_NO_7_P | M+H | ↑**, √ | ↓, √ | ↓*, √ | ↓*, √ | ↓, √ | ↓, √ | ↓, √ |
| 45 | 562.3154 | 8.8 | LysoPC(16:0/0:0) | C_24_H_50_NO_7_P | M-H+HCOONa | ↑ | ↓, √ | ↓, √ | ↑, √ | ↑*, √ | ↓, √ | ↑, √ |
| 46 | 482.3236 | 9.1 | LysoPC(15:0/0:0) | C_23_H_48_NO_7_P | M+H | ↑, √ | ↓, √ | ↓, √ | ↓, √ | ↑, √ | ↓, √ | ↑, √ |
| 47 | 524.3703 | 10.0 | LysoPC(0:0/18:0) | C_26_H_54_NO_7_P | M+H | ↑, √ | ↓, √ | ↓*, √ | ↓ | ↑, √ | ↓ | ↑, √ |
| 48 | 564.3317 | 9.3 | LysoPC(18:2(9Z,12Z)/0:0) | C_26_H_50_NO_7_P | M+FA-H | ↑, √ | ↓, √ | ↓*, √ | ↑, √ | ↑, √ | ↓*, √ | ↑*, √ |
| 49 | 572.3704 | 10.1 | LysoPC(22:4(7Z,10Z,13Z,16Z)/0:0) | C_30_H_54_NO_7_P | M+H | ↑ | ↑ | ↓, √ | ↑ | ↓ | ↓ | ↑* |
| 50 | 522.3559 | 10.0 | LysoPC(0:0/18:1(9Z)) | C_26_H_52_NO_7_P | M+H | ↑, √ | ↓, √ | ↓*, √ | ↓, √ | ↑, √ | ↓, √ | ↑, √ |
| 51 | 836.6149 | 7.7 | PC(24:1(15Z)/14:1(9Z)) | C_46_H_88_NO_8_P | M+Na | ↓, √ | ↑ | ↑* | ↑ | ↓, √ | ↑* | ↑, √ |
| 52 | 454.2921 | 9.3 | LysoPE(16:0/0:0) | C_21_H_44_NO_7_P | M+H | ↓, √ | ↑ | ↓, √ | ↓ | ↓, √ | ↑, √ | ↓, √ |
| 53 | 476.2781 | 9.1 | LysoPE(18:2(9Z,12Z)/0:0) | C_23_H_44_NO_7_P | M-H | ↑ | ↓ | ↓, √ | ↑*, √ | ↑, √ | ↓, √ | ↑**, √ |
| 54 | 510.3547 | 10.6 | LysoPE(20:0/0:0) | C_25_H_52_NO_7_P | M+H | ↑, √ | ↓, √ | ↓, √ | ↓, √ | ↑, √ | ↓, √ | ↑, √ |
