# Supplementary material for: Protective effects of Descurainia sophia seeds extract and its fractions on pulmonary edema by untargeted urine and serum metabolomics strategy
Source: Front Pharmacol. 2023 Feb 14;14:1080962. doi: 10.3389/fphar.2023.1080962 (PMC9971919; doi:10.3389/fphar.2023.1080962)
Supplement: Supplementary file 2 [file DataSheet3.docx]

**Supplementary Material 3**

**TABLE S1** Identified biomarkers related to PE/DS-Pol/ DS-Oli/DS-FG/DS-FA/DS-FO in urine. “√” represents the biomarkers found between the treatment group *vs.* PE. No “√” represents the absence of the biomarkers between the treatment group *vs.* PE.

↓ down-regulation; ↑ up-regulation (*P < 0.05, **P < 0.01).

**^a^** Change trend of PE group compared with NC group.

**^b^** Change trend of DS, DS-Pol, DS-Oli, DS-FG, DS-FA and DS-FO groups compared with PE group, respectively.

|  | **Detected**  **m/z** | **Rt**  **(min)** | **Metabolites** | **Formula** | **Adduct** | | **PE**  ***vs.* NC^a^** | **DS^b^**  ***vs.* PE** | **DS-Pol^b^**  ***vs.* PE** | **DS-Oli^b^**  ***vs.* PE** | **DS-FG^b^**  ***vs.* PE** | **DS-FA^b^**  ***vs.* PE** | **DS-FO^b^**  ***vs.* PE** |
| --- | --- | --- | --- | --- | --- | --- | --- | --- | --- | --- | --- | --- | --- |
| 1 | 248.0314 | 8.5 | Pyridoxal 5'-phosphate | C_8_H_10_NO_6_P | | M+H | ↑, √ | ↑, √ | ↓, √ | ↓, √ | ↓, √ | ↑**, √ | ↑, √ |
| 2 | 170.0813 | 0.9 | Pyridoxine | C_8_H_11_NO_3_ | | M+H | ↓**, √ | ↑*, √ | ↑, √ | ↑**, √ | ↑, √ | ↑*, √ | ↑, √ |
| 3 | 168.0653 | 1.1 | Pyridoxal | C_8_H_9_NO_3_ | | M+H | ↓, √ | ↑*, √ | ↑, √ | ↑, √ | ↓, √ | ↑, √ | ↓, √ |
| 4 | 182.0456 | 1.3 | 4-Pyridoxic acid | C_8_H_9_NO_4_ | | M-H | ↓, √ | ↑, √ | ↑ | ↑**, √ | ↑, √ | ↑*, √ | ↑, √ |
| 5 | 124.0072 | 1.1 | Taurine | C_2_H_7_NO_3_S | | M-H | ↑**, √ | ↓, √ | ↓*, √ | ↓*, √ | ↓**, √ | ↓**, √ | ↓**, √ |
| 6 | 166.0177 | 1.1 | L-Cysteine | C_3_H_7_NO_2_S | | M+FA-H | ↑**, √ | ↓*, √ | ↓**, √ | ↓**, √ | ↓**, √ | ↓, √ | ↓**, √ |
| 7 | 253.0505 | 19.9 | 5-L-Glutamyl-taurine | C_7_H_14_N_2_O_6_S | | M-H | ↓, √ | ↑**, √ | ↓, √ | ↑ | ↑, √ | ↑, √ | ↑, √ |
| 8 | 134.0598 | 6.3 | Indoxyl | C_8_H_7_NO | | M+H | ↑, √ | ↓, √ | ↓*, √ | ↓, √ | ↓*, √ | ↓, √ | ↓*, √ |
| 9 | 212.0025 | 6.1 | Indoxyl sulfate | C_8_H_7_NO_4_S | | M-H | ↑, √ | ↓, √ | ↓*, √ | ↓, √ | ↓*, √ | ↓, √ | ↓*, √ |
| 10 | 176.0702 | 7.5 | Indoleacetic acid | C_10_H_9_NO_2_ | | M+H | ↑*, √ | ↓**, √ | ↓**, √ | ↓**, √ | ↓**, √ | ↓**, √ | ↓**, √ |
| 11 | 206.0807 | 11.9 | 5-Methoxyindoleacetate | C_11_H_11_NO_3_ | | M+H | ↑, √ | ↓**, √ | ↓**, √ | ↓** | ↓**, √ | ↓, √ | ↓** |
| 12 | 208.0598 | 6.1 | 4-(2-Aminophenyl)-2,4-dioxobutanoic acid | C_10_H_9_NO_4_ | | M+H | ↑*, √ | ↓, √ | ↓, √ | ↓**, √ | ↓, √ | ↓*, √ | ↓, √ |
| 13 | 265.1174 | 6.7 | Acetyl-N-formyl-5-methoxykynurenamine | C_13_H_16_N_2_O_4_ | | M+H | ↑ | ↓**, √ | ↓**, √ | ↓**, √ | ↓**, √ | ↓**, √ | ↓**, √ |
| 14 | 188.0350 | 4.5 | Kynurenic acid | C_10_H_7_NO_3_ | | M-H | ↑**, √ | ↑, √ | ↓, √ | ↓, √ | ↓, √ | ↓, √ | ↓**, √ |
| 15 | 206.0446 | 1.7 | Xanthurenic acid | C_10_H_7_NO_4_ | | M+H | ↑**, √ | ↓, √ | ↓*, √ | ↓**, √ | ↓**, √ | ↓**, √ | ↓**, √ |
| 16 | 175.0244 | 1.3 | Pyruvic acid | C_3_H_4_O_3_ | | 2M-H | ↑**, √ | ↓**, √ | ↓** | ↓** | ↓*, √ | ↓** | ↓*, √ |
| 17 | 173.0091 | 1.3 | cis-Aconitic acid | C_6_H_6_O_6_ | | M-H | ↑**, √ | ↓**, √ | ↓, √ | ↓**, √ | ↓**, √ | ↓**, √ | ↓*, √ |
| 18 | 191.0198 | 1.3 | Citric acid | C_6_H_8_O_7_ | | M-H | ↑, √ | ↓*, √ | ↓, √ | ↓, √ | ↑, √ | ↓, √ | ↑, √ |
| 19 | 117.0192 | 1.3 | Succinic acid | C_4_H_6_O_4_ | | M-H | ↑, √ | ↓*, √ | ↓, √ | ↓, √ | ↓, √ | ↓, √ | ↑, √ |
| 20 | 189.0032 | 8.7 | Oxalosuccinic acid | C_6_H_6_O_7_ | | M-H | ↑, √ | ↓**, √ | ↓**, √ | ↓**, √ | ↓**, √ | ↓**, √ | ↓**, √ |
| 21 | 130.0499 | 1.1 | L-Glutamic acid | C_5_H_9_NO_4_ | | M+H-H2O | ↑, √ | ↓, √ | ↑ | ↓, √ | ↓, √ | ↓, √ | ↓, √ |
| 22 | 175.1186 | 1.1 | L-Arginine | C_6_H_14_N_4_O_2_ | | M+H | ↑, √ | ↑, √ | ↓ | ↑ | ↓*, √ | ↑ | ↓, √ |
| 23 | 129.0658 | 1.1 | L-Glutamine | C_5_H_10_N_2_O_3_ | | M+H-H2O | ↑**, √ | ↓, √ | ↓, √ | ↓, √ | ↓*, √ | ↓, √ | ↓*, √ |
| 24 | 114.0664 | 0.9 | Creatinine | C_4_H_7_N_3_O | | M+H | ↑**, √ | ↓, √ | ↓*, √ | ↓, √ | ↓**, √ | ↓, √ | ↓**, √ |
| 25 | 132.0766 | 1.1 | Creatine | C_4_H_9_N_3_O_2_ | | M+H | ↑**, √ | ↓*, √ | ↓, √ | ↓**, √ | ↓**, √ | ↓, √ | ↓**, √ |
| 26 | 231.1336 | 1.1 | L-Proline | C_5_H_9_NO_2_ | | 2M+H | ↑**, √ | ↓*, √ | ↓**, √ | ↓**, √ | ↓*, √ | ↓**, √ | ↓*, √ |
| 27 | 131.1290 | 0.9 | Agmatine | C_5_H_14_N_4_ | | M+H | ↓** | ↑, √ | ↑, √ | ↑, √ | ↑, √ | ↑, √ | ↑, √ |
| 28 | 120.0808 | 1.1 | Tyramine | C_8_H_11_NO | | M+H-H2O | ↑*, √ | ↑, √ | ↓ | ↓, √ | ↓, √ | ↓, √ | ↓*, √ |
| 29 | 181.0505 | 3.9 | Homovanillic acid | C_9_H_10_O_4_ | | M-H | ↑*, √ | ↑ | ↓, √ | ↓, √ | ↓, √ | ↓, √ | ↓*, √ |
| 30 | 134.0610 | 6.3 | Dopamine | C_8_H_11_NO_2_ | | M-H20-H | ↓**, √ | ↓ | ↓ | ↓ | ↓** | ↑, √ | ↓, √ |
| 31 | 194.0456 | 8.9 | Dopaquinone | C_9_H_9_NO_4_ | | M-H | ↓, √ | ↑**, √ | ↑, √ | ↑, √ | ↑, √ | ↑**, √ | ↑, √ |
| 32 | 147.0450 | 8.9 | Homovanillin | C_9_H_10_O_3_ | | M-H20-H | ↓ | ↑**, √ | ↑, √ | ↑ | ↑*, √ | ↑, √ | ↑, √ |
| 33 | 164.0715 | 8.1 | Epinephrine | C_9_H_13_NO_3_ | | M-H20-H | ↑ | ↓, √ | ↓, √ | ↓, √ | ↓, √ | ↑*, √ | ↓, √ |
| 34 | 153.0192 | 7.3 | Gentisic acid | C_7_H_6_O_4_ | | M-H | ↓, √ | ↑*, √ | ↑, √ | ↑, √ | ↑, √ | ↑*, √ | ↑, √ |
| 35 | 121.0649 | 8.9 | Tyrosol | C_8_H_10_O_2_ | | M+H-H2O | ↓, √ | ↑**, √ | ↑ | ↑ | ↑* | ↑, √ | ↑, √ |
| 36 | 112.0504 | 0.9 | Cytosine | C_4_H_5_N_3_O | | M+H | ↑ | ↓ | ↓*, √ | ↓**, √ | ↓**, √ | ↓, √ | ↓**, √ |
