# Supplementary material for: Protective effects of Descurainia sophia seeds extract and its fractions on pulmonary edema by untargeted urine and serum metabolomics strategy
Source: Front Pharmacol. 2023 Feb 14;14:1080962. doi: 10.3389/fphar.2023.1080962 (PMC9971919; doi:10.3389/fphar.2023.1080962)
Supplement: Supplementary file 3 [file DataSheet2.docx]

**Supplementary Material 2**

**TABLE 1** All values of R^2^X, R^2^Y, Q^2^ of seven OPLS-DA models were listed.

|  | group | R^2^X | R^2^Y | Q^2^ |
| --- | --- | --- | --- | --- |
| OPLS-DA (urine) | PE *vs.* NC | 0.66 | 0.994 | 0.974 |
|  | DS *vs.* PE | 0.741 | 0.996 | 0.947 |
|  | DS-Pol *vs.* PE | 0.917 | 1 | 0.963 |
|  | DS-Oli *vs.* PE | 0.868 | 1 | 0.974 |
|  | DS-FG *vs.* PE | 0.764 | 0.996 | 0.941 |
|  | DS-FA *vs.* PE | 0.913 | 1 | 0.964 |
|  | DS-FO *vs.* PE | 0.839 | 1 | 0.904 |
| OPLS-DA (serum) | PE *vs.* NC | 0.572 | 0.995 | 0.967 |
|  | DS *vs.* PE | 0.336 | 0.994 | 0.935 |
|  | DS- Pol *vs.* PE | 0.405 | 0.997 | 0.97 |
|  | DS-Oli *vs.* PE | 0.43 | 0.989 | 0.948 |
|  | DS-FG *vs.* PE | 0.466 | 0.985 | 0.937 |
|  | DS-FA *vs.* PE | 0.472 | 0.989 | 0.948 |
|  | DS-FO *vs.* PE | 0.603 | 0.998 | 0.897 |
