# Supplementary material for: Protective effects of Descurainia sophia seeds extract and its fractions on pulmonary edema by untargeted urine and serum metabolomics strategy
Source: Front Pharmacol. 2023 Feb 14;14:1080962. doi: 10.3389/fphar.2023.1080962 (PMC9971919; doi:10.3389/fphar.2023.1080962)
Supplement: Supplementary file 4 [file DataSheet1.DOCX]

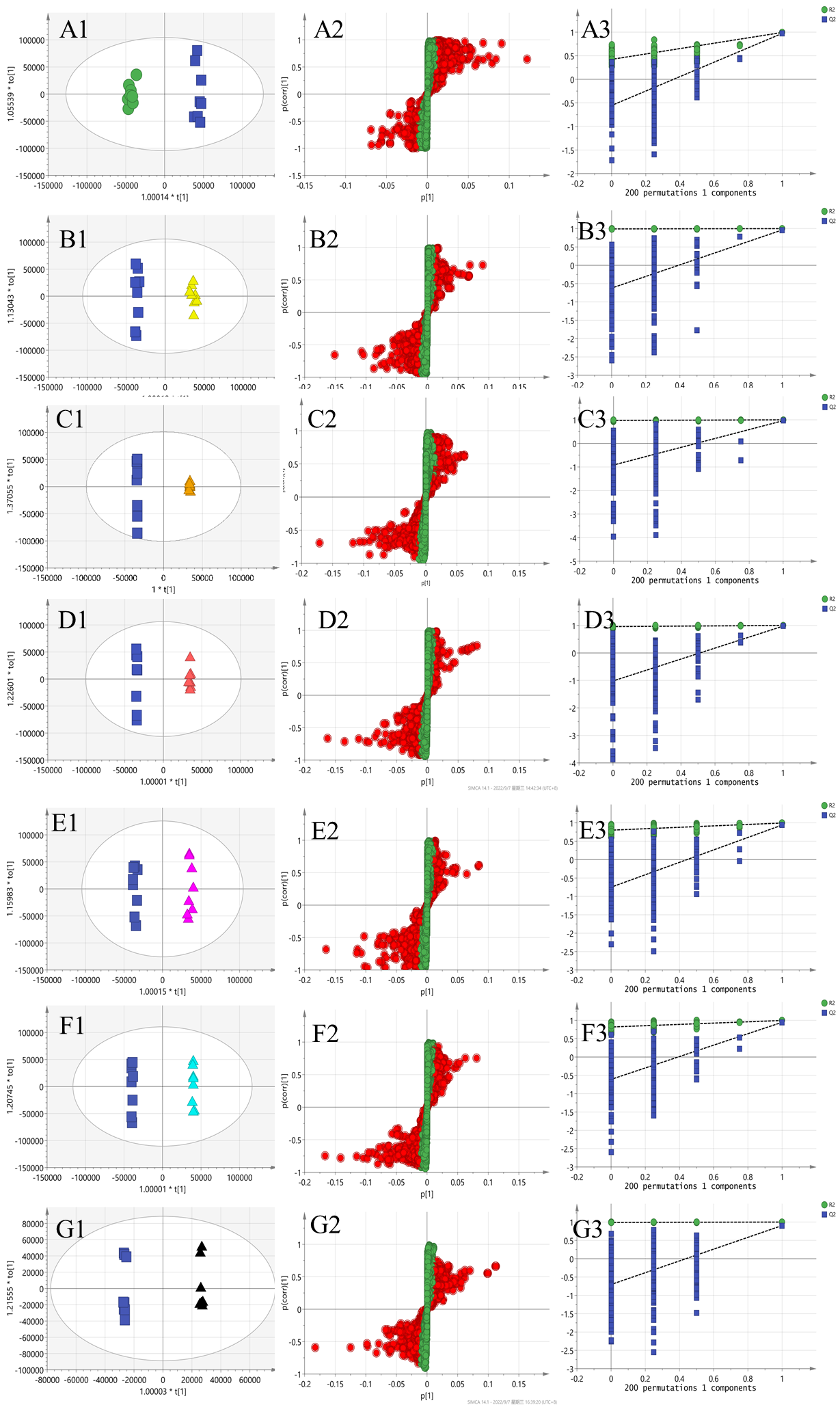
Supplementary Material 1

**Figure 1** OPLS-DA score plots, S-plots, validation plots obtained from NC, DS, DS-Pol, DS-Oli, DS-FG, DS-FA, DS-FO *vs.* PE in urine (A1-A3/B1-B3/C1-C3/D1-D3/E1-E3/F1-F3/G1-G3 ). The colors and shapes of symbols in the above pictures are align with PCA score plots.


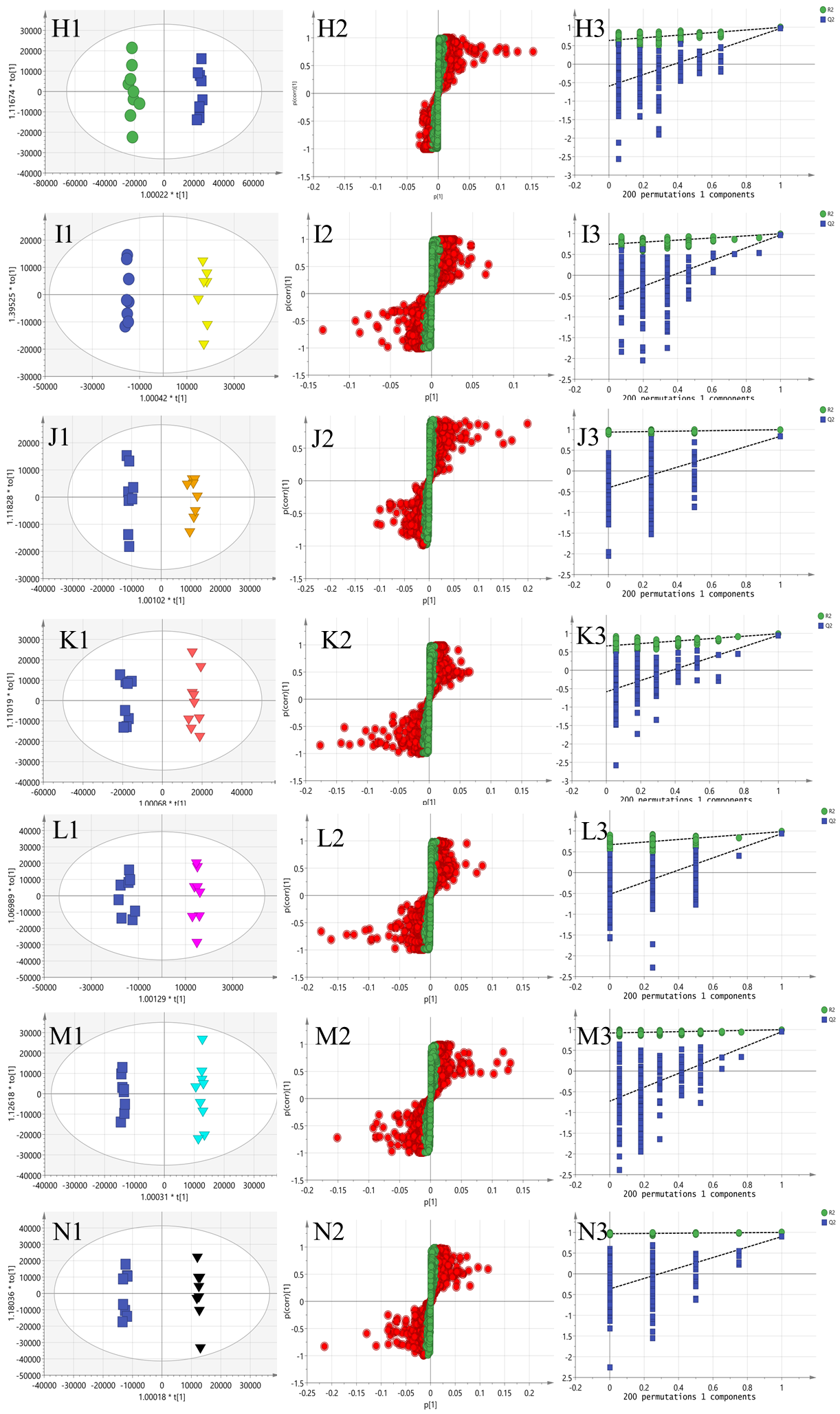


**Figure 2** OPLS-DA score plots, S-plots, validation plots obtained from NC, DS, DS-Pol, DS-Oli, DS-FG, DS-FA, DS-FO *vs.* PE in serum (H1-H3/I1-I3/J1-J3/ K1-K3/L1-L3/M1-M3/N1-N3). The colors and shapes of symbols in the above pictures are align with PCA score plots.
